# Nutritional status and changes in muscle and adipose tissue determined by computed tomography as predictors of mortality in hospitalized patients

**DOI:** 10.1590/0100-3984.2024.0026

**Published:** 2024-11-07

**Authors:** Bruna Lúcia de Mendonça Soares, Nataly Maria de Mendonça Soares, Maria Goretti Pessoa de Araújo Burgos, Ilma Kruze Grande de Arruda

**Affiliations:** 1 Graduate Program in Nutrition, Department of Nutrition, Universidade Federal de Pernambuco (UFPE), Recife, PE, Brazil; 2 Hospital da Restauração Governador Paulo Guerra, Recife, PE, Brazil; 3 Faculdade Integrada Tiradentes, Jaboatão dos Guararapes, PE, Brazil

**Keywords:** Body composition, Hospitalization, Malnutrition, Obesity paradox, Composição corporal, Hospitalização, Desnutrição, Paradoxo da obesidade

## Abstract

The aim of the present study was to investigate whether nutritional status and changes in muscle and adipose tissue determined by computed tomography are predictors of mortality in hospitalized patients. This was a prospective cohort study involving patients ≥ 20 years of age hospitalized in a public hospital. Sociodemographic and clinical variables were collected from electronic medical records. Nutritional variables were determined. All patients were followed prospectively until the hospital outcome, which could be discharge or death. Body composition was defined from computed tomography images, with the identification of myopenia, myosteatosis, and myopenic obesity. The sample comprised 542 patients. The mortality rate was 10.7% (95% CI: 6.4-15.0%). The independent predictors of mortality were malnutrition, assessed with the subjective global assessment (hazard ratio: 4.18; 95% CI: 1.01-17.22; *p* = 0.047), and myopenic obesity (hazard ratio: 2.82; 95% CI: 1.11-7.20; *p* = 0.029). The findings of the present study add to the limited evidence in the literature that body composition is associated with outcomes in hospitalized patients.

## Abstract (pt)

O objetivo do presente estudo foi investigar se o estado nutricional e as alterações nos tecidos muscular e adiposo, determinados pela tomografia computadorizada, são preditores de mortalidade em pacientes hospitalizados. Foi realizado um estudo de coorte prospectivo envolvendo pacientes com idade ≥ 20 anos, internados em um hospital público. Variáveis sociodemográficas e clínicas foram coletadas do prontuário eletrônico e variáveis nutricionais foram aferidas. Todos os pacientes foram acompanhados prospectivamente até o desfecho hospitalar, que poderia ser alta ou óbito. A composição corporal foi definida com base em imagens de tomografia computadorizada, com determinação da presença de miopenia, mioesteatose e obesidade visceral miopênica. A amostra foi composta por 542 pacientes. A mortalidade foi encontrada em 10,7% (IC 95%: 6,4-15,0%). Os preditores independentes de mortalidade foram a desnutrição avaliada pela avaliação subjetiva global (risco relativo: 4,18; IC 95%: 1,01-17,22; *p* = 0,047) e a presença de obesidade visceral miopênica (risco relativo: 2,82; IC 95%: 1,11-7,20; *p* = 0,029). Os achados do presente estudo somam-se às limitadas evidências publicadas de que a composição corporal está associada a desfechos em pacientes hospitalizados.

## INTRODUCTION

Nutritional status is a determinant of clinical and surgical outcomes in hospitalized patients. In this context, the assessment of body composition can reveal muscle loss associated with malnutrition, as well as a predominance of subcutaneous or visceral adipose tissue (VAT), which is not easily detectable during the physical examination, by calculating the body mass index (BMI), or by other conventional anthropometric measures^**(1-3)**^.

Although various methods can be used to estimate body composition, the analysis of computed tomography (CT) images is considered the gold standard for the determination of skeletal muscle mass, as well as the quantity/ distribution of adipose tissue and the specific radiodensity of tissues^**(3-5)**^.

Low skeletal muscle radiodensity, which is known as myosteatosis, reflects the intramuscular infiltration of fat and is a radiologically defined prognostic marker of poor muscle quality. Myosteatosis can directly affect patient survival, as a greater presence of fat in skeletal muscle exerts a greater negative impact on health outcomes^**(4,6)**^.

Although the role of muscle mass in outcomes among hospitalized patients is well established in the literature, studies have mainly used the skeletal muscle mass index to predict the risk of complications and mortality in this population without considering other body components determined by CT, such as the presence of myosteatosis and visceral obesity^**(5-8)**^.

The associations that clinical outcomes, such as mortality, have with adiposity, myopenia (low muscle mass), and myosteatosis need to be explored and understood. Therefore, the aim of the present study was to investigate whether nutritional status and changes in muscle and adipose tissue determined by CT are predictors of mortality.

## METHOD

This was a prospective cohort study, with data collection between February 2021 and July 2022 at a tertiary public hospital. The study was approved by the local human research ethics committee (Reference no. 38669820.8.0000.5208). Patients were considered eligible if they were ≥ 20 years of age, had been admitted to a medical or surgical ward, and had undergone CT during hospitalization. Patients in palliative treatment or end-oflife care were excluded.

Sociodemographic characteristics (age and sex) and clinical data (clinical diagnosis, comorbidities, and serum albumin) were extracted from electronic medical records. Patients ≥ 60 years of age were classified as older adults. After the initial assessments, all patients were followed prospectively until the hospital outcome, which could be discharge or death.

Nutritional status was assessed by using anthropometric measures, a subjective assessment, and serum albumin levels. The classification of nutritional status based on serum albumin levels was performed as recommended by Blackburn et al.^**(9)**^. Current weight was determined by using a scale with a capacity of 200 kg and a precision of 0.1 kg (Filizola, São Paulo, Brazil). Height was measured using a stadiometer coupled to the scale. For patients with difficulty remaining in the standing position, height was estimated based on knee height, as described by Chumlea et al.^**(10)**^.

Weight and height were used for the calculation of the BMI and for the classification of nutritional status^**(11,12)**^. Calf circumference (CC) was measured with a nonelastic tape (Sanny, São Paulo, Brazil) with the patient sitting and the legs at a 90° angle. The cutoff point adopted for low CC was ≤ 33 cm for women and ≤ 34 cm for men^**(13)**^.

A score of ≥ 3 on the 2002 nutritional risk screening (NRS-2002) tool was considered indicative of nutritional risk^**(14)**^. For patients at risk of malnourishment, the subjective global assessment (SGA) was used for the diagnosis of malnutrition^**(15)**^. The Strength, Assistance in walking, Rise from a chair, Climb stairs, and Falls (SARC-F) questionnaire was used in order to screen for the risk of sarcopenia, with a score of ≥ 4 points indicating the presence of risk^**(16)**^.

Abdominal CT images captured for the purposes of routine medical investigations were used to quantify crosssectional areas of muscle and adipose tissue. Two consecutive images of the region of the cross-section marked on the third lumbar vertebra were analyzed by a single assessor using the OsiriX Lite program, version 13.0.0 (Pixmeo SARL, Bernex, Switzerland). The average of the two measurements was considered for analysis.

The area (cm^2^) of the tissues was obtained by measuring radiodensity. For such, predetermined thresholds were used^**(17,18)**^: -29 to 150 Hounsfield units (HU) for skeletal muscle mass (SMM) and the psoas muscle (PM); -50 to -150 HU for VAT; and -30 to -190 HU for subcutaneous adipose tissue (SAT) and intramuscular adipose tissue (IMAT). The extent of total adipose tissue (TAT; cm^2^) was calculated as the sum of the VAT, SAT, and IMAT values. The area of the respective tissues was normalized by the height squared of the patients and reported as indices (cm^2^/m^2^): SMMI, PMI, VATI, SATI, IMATI, and TATI.

Loss of muscle mass (myopenia) was defined as an SMMI ≤ 38.5 cm^2^/m^2^ for women and ≤ 52.5 cm^2^/m^2^ for men^**(^19^)**^. The VATI cutoff point proposed by Baggerman et al.^**(^20^)**^ was used for the identification of visceral obesity: ≥ 38.7 cm^2^/m^2^ for men and ≥ 24.9 cm^2^/m^2^ for women. Myopenic obesity was defined as the concomitant presence of myopenia and visceral obesity, as determined by CT. The mean muscle attenuation of the entire muscle area in the third lumbar vertebra region (expressed in HU) was determined in order to estimate the infiltration of fat into skeletal muscle (myosteatosis). Therefore, low mean muscle attenuation-indicative of myosteatosis-was recorded when the attenuation was < 41 HU in individuals with a BMI ≤ 24.9 kg/m^2^ or < 33 HU in individuals with a BMI ≥ 25 kg/m^2**(^21^)**^.

Statistical analysis was performed with the Statistical Package for the Social Sciences, version 16.0 (SPSS Inc., Chicago, IL, USA). The Kolmogorov-Smirnov test was used in order to determine the distribution of continuous variables. Those with a parametric distribution are expressed as mean and standard deviation and those with a nonparametric distribution are expressed as median and interquartile range. Comparisons were performed with parametric or nonparametric tests. Univariate comparisons of the results of the time to the event (death) were performed with Kaplan-Meier curves and log-rank tests. Multivariate adjusted hazard ratios were estimated from Cox proportional risk models. To adjust for possible confounding factors, all variables associated with the outcome, hospital stay, or readmission were included in the regression with sex, age, and diagnosis. Values of *p* < 0.05 were considered indicative of statistical significance.

## RESULTS

**Figure 1:**
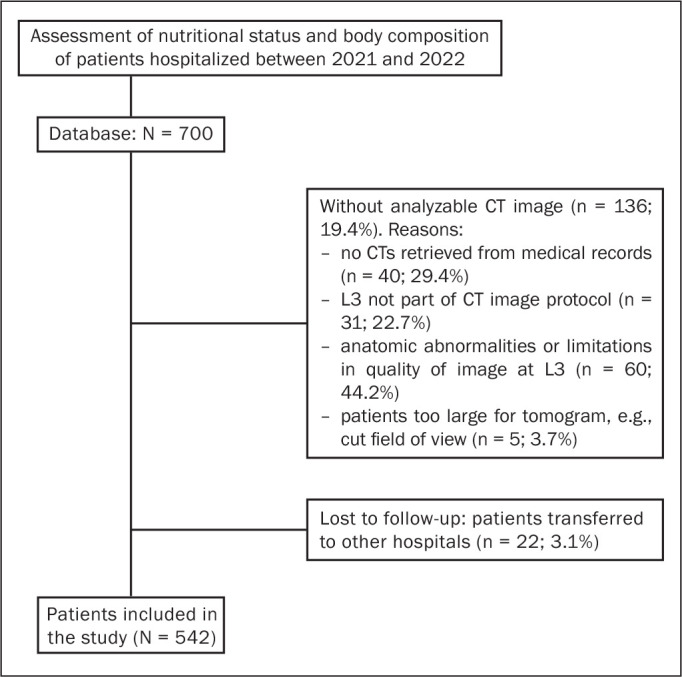
Flow diagram of the study sample.

Seven hundred patients were included in the database. However, 158 (22.6%) were excluded: 136 because of a lack of analyzable CT images; and 22 because they were lost to follow-up. Therefore, the final sample comprised 542 patients (Figure 1). The mean age was 53.5 ± 18.5 years. In the sample, there was a predominance of men, of patients admitted to the surgical ward, and of patients with neoplasms (Table 1).

**Table 1:** Demographic, clinical, and nutritional characteristics of the sample (N = 542).

Variable	n (%)	95% Cl
Sex		
Male	329 (60.7)	53.9-67.5
Female	213 (39.3)	32.5-46.1
Age group		
Adults	307 (56.6)	49.8-63.5
Older adults	235 (43.4)	36.5-50.2
Diagnostic categories		
Neurological	39 (7.2)	3.6-10.8
Hepatic	29 (5.4)	2.2-8.5
Trauma	106 (19.6)	14.1-25.1
Neoplasms	247 (45.6)	38.7-52.5
Vascular	15 (2.8)	0.5-5.0
Gastrointestinal	93 (17.2)	11.9-22.4
Other^*^	13 (2.4)	0.3-4.5
Type of hospitalization		
Clinical	138 (25.5)	19.4-31.5
Surgical	404 (74.5)	68.5-80.6
Albumin^†^		
Malnourished	336 (65.5)	58.9-72.1
Normal weight	177 (34.5)	27.9-41.1
SARC-F, risk of sarcopenia		
Present	252 (46.5)	39.6-53.4
Absent	290 (53.5)	46.6-60.4
NRS-2002, nutritional risk		
Present	354 (65.3)	58.7-71.9
Absent	188 (34.7)	28.1-41.3
SGA, malnutrition		
Present	322 (59.4)	52.6-66.2
Absent	220 (40.6)	33.8-47.4
BMI, nutritional status		
Underweight	162 (29.9)	23.5-36.2
Normal weight	227 (41.9)	35.0-48.7
Excess weight	153 (28.2)	22.0-34.5
CC^‡^. muscle mass		
Low	265 (53.6)	46.7-60.6
Normal	229 (46.4)	39.4-53.3
Myopenia		
Present	260 (48.0)	41.0-54.9
Absent	282 (52.0)	45.1-59.0
Visceral obesity		
Present	226 (41.7)	34.9-48.5
Absent	316 (58.3)	51.5-65.1
Myopenic obesity		
Present	88(16.2)	11.1-21.3
Absent	454 (83.8)	78.7-88.9
Myosteatosis		
Present	396 (73.1)	66.9-79.2
Absent	146 (26.9)	20.8-33.1

* Cardiovascular, infectious-parasitic, and dermatological diseases.

† n = 513.

‡ n = 494.

Although 41.9% of patients were classified as normal weight based on the BMI, the proportions of patients at nutritional risk, at risk for sarcopenia, and with malnutrition, as assessed with the NRS-2002, SARC-F, and SGA, were higher (65.3%, 46.5%, and 59.4%, respectively). The CC measurements indicated that 53.6% of patients had reduced muscle mass, and low serum albumin concentrations suggested that 65.5% of individuals had compromised nutritional status. Myopenia and visceral obesity were found in 48.0% and 41.7% of the sample, respectively. However, myopenic obesity (situation in which both conditions occur simultaneously) was observed in only 16.2%. Myosteatosis (infiltration of fat in skeletal muscle) was found in 73.1%.

**Figure 2:**
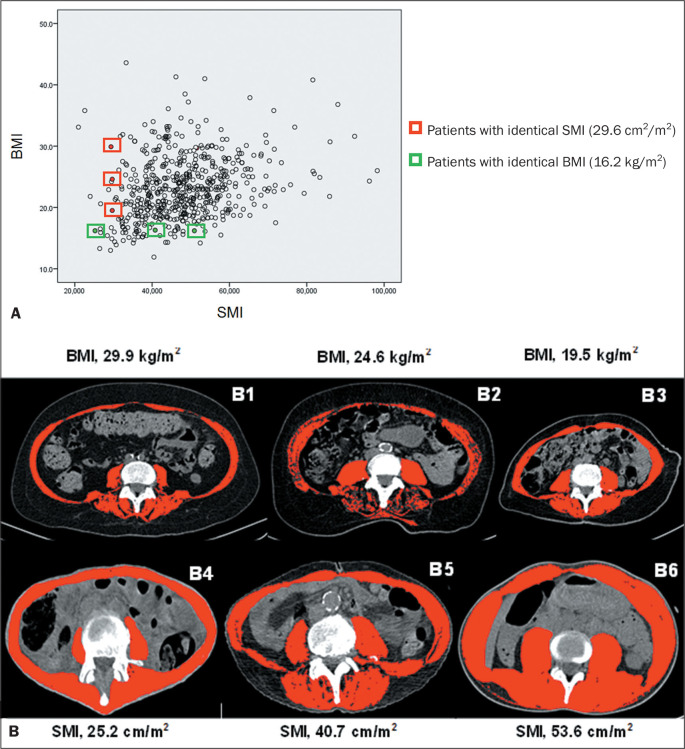
A: Scatter plot highlighting the relationship and variation between SMI and BMI (N = 564; Pearson’s r = 0.26; *p* = 0.000). Green boxes indicate female patients with identical BMIs (16.2 kg/m^2^), and red boxes indicate male patients with identical SMIs (29.6 cm^2^/m^2^). B: Axial CT images of the region of the third lumbar vertebra with skeletal muscle indicated in red (-29 to 150 HU). Panels B1-3 highlight the variation in BMI among the male patients with identical SMIs (points corresponding to these images are indicated with red boxes in panel A). Panels B4-6 highlight the variation in SMI among the female patients with identical BMIs (points corresponding to these images are indicated with green boxes in panel A).

Figure 2 exemplifies, through a scatter plot, the relationship and variation in the skeletal muscle index (SMI) and BMI (Figure 2A). The figure also shows CT images to better highlight the differences and similarities between muscle mass and BMI (Figure 2B). As can be seen in the figure, three female patients, despite having the same BMI, in the underweight range (16.2 kg/m^2^), presented significantly different amounts of muscle mass, one being classified as having myopenia and the other two being classified as having adequate amounts of skeletal muscle mass.

The opposite was also observed; that is, a low amount of muscle mass can be present in all BMI ranges. As shown in Figure 2, three male patients, all with myopenia (identical SMI of 29.6 cm^2^/m^2^), had different BMIs. One, despite being overweight, was myopenic. And another (elderly) patient, despite being underweight, had the same SMI as the two other patients.

**Figure 3:**
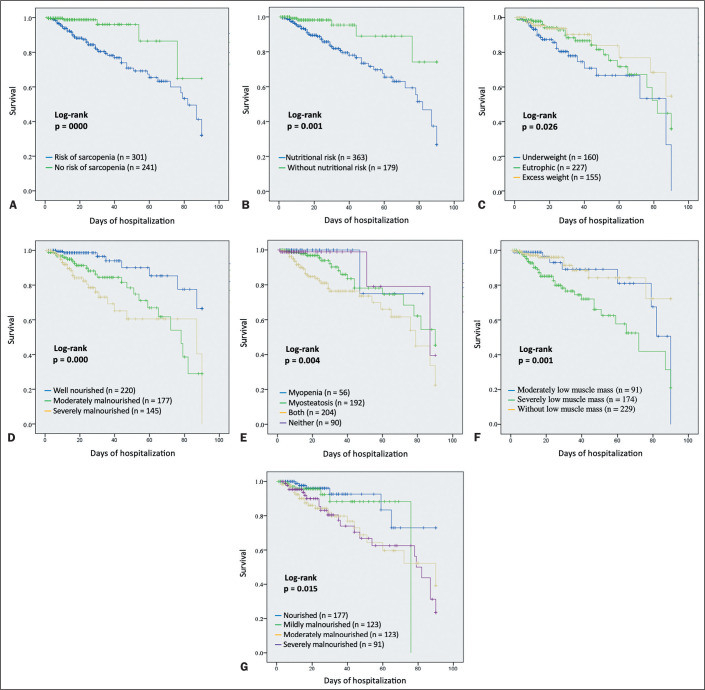
Kaplan-Meier curves of all-cause mortality based on nutritional status and body composition. A: Kaplan-Meier curves categorized by the risk of sarcopenia, as defined with the SARC-F questionnaire. B: Kaplan-Meier curves categorized by nutritional risk, as defined with the NRS-2002 tool. C: Kaplan-Meier curves categorized by BMI. D: Kaplan-Meier curves categorized by nutritional status, as defined with the SGA. E: Kaplan-Meier curves categorized by myopenia and myosteatosis. F: Kaplan-Meier curves categorized by reduction in muscle mass, as quantified by the CC. G: Kaplan-Meier curves categorized by nutritional status, as defined by the serum albumin level. Unadjusted mortality rates were calculated by using Kaplan-Meier methods and were compared by using log-rank tests.

The outcome discharge occurred in 89.3% of the patients (95% CI: 8.0-93.6%), wher eas in-hospital death occurred in 10.7% (95% CI: 6.4-15.0%). The KaplanMeier curves illustrate the incidence of death with respect to nutritional status and body composition (Figure 3). The incidence of mortality was higher in patients screened for sarcopenia; in those with nutritional risk, low BMI, or low muscle mass based on the CC; and in those with severe malnutrition assessed on the basis of the SGA and serum albumin level. The mortality rate was highest among the patients with concomitant myopenia and myosteatosis.

The results of the Cox regression analysis for clinical and nutritional factors associated with mortality are shown in Tables 2 and 3. The patients who died were older, with a median age of 63.5 years (interquartile range [IQR]: 46.0- 71.0 years), compared with 54.0 years (IQR: 40.0-66.0 years) for those who were discharged (*p* = 0.013); had a longer median hospital stay-24.0 days (IQR: 11.0-47.0 days) versus 13.0 days (IQR: 6.5-25.0 days) among the discharged patients (*p* = 0.001); were all admitted to the surgical ward; all had neoplasms; and had a lower median concentration of serum albumin-2.4 g/dL (IQR: 2.1-2.8 g/dL), compared with 3.1 g/dL (IQR: 2.5-3.8 g/dL) for those who were discharged (*p* = 0.000). Therefore, all of these variables were associated with an increased risk of death.

**Table 2:** Associations of demographic, clinical, and nutritional variables with in-hospital death (N = 542).

Variable	Total (n)	Death n (%)	HR (95% Cl)	P^*^
Sex				0.588
Male	329	36 (10.9)		
Female	213	22 (10.3)	0.86 (0.50-1.47)	
Age				0.005
< 60 years	307	24 (7.8)		
> 60 years	235	34 (14.5)	2.14 (1.26-3.62)	
Type of admission				0.001
Surgical	404	44 (10.9)		
Clinical	138	14 (10.1)	2.69 (1.46-4.96)	
Neoplasm				0.050
Present	247	27 (10.9)		
Absent	295	31 (10.5)	1.78 (1.00-2.91)	
Albumin, malnutrition				0.015
Present	336	52 (15.5)		
Absent	177	6(3.4)	2.87 (1.23-6.71)	
SARC-F, risk of sareopenia				0.001
Present	252	49 (19.4)		
Absent	290	9(3.1)	3.50 (1.70-7.22)	
NRS-2002, nutritional risk				0.003
Present	354	52 (14.7)		
Absent	188	6 (3.2)	3.68 (1.58-8.58)	
SGA, malnutrition				0.000
Present	322	50 (15.5)		
Absent	220	8 (3.6)	4.48 (2.11-9.48)	
Low BMI				0.009
Present	161	24 (14.9)		
Absent	380	34 (8.7)	2.02 (1.19-3.44)	
Low CC^†^				0.005
Present	265	42 (15.8)		
Absent	229	11 (4.8)	2.59 (1.33-5.04)	
Myopenia				0.009
Present	260	37 (14.2)		
Absent	282	21 (7.4)	2.04 (1.19-3.51)	
Visceral obesity				0.428
Present	226	27 (11.9)		
Absent	316	31 (9.8)	1.23 (0.73-2.06)	
Myopenic obesity				0.005
Present	88	19 (21.6)		
Absent	454	39 (8.6)	2.19 (1.26-3.79)	
Myosteatosis				0.001
Present	396	54 (13.6)		
Absent	146	4(2.7)	5.60(1.99-15.76)	

HR, hazard ratio.

* Cox regression.

† n = 514.

**Table 3:** Multivariate analysis of potential risk factor for death.

Independent variables	Unadjusted			Adjusted		
	HR	95% Cl	P^*^	HR	95% Cl	P^*^
Albumin, malnutrition						
<3.5 g/dL >3.5 g/dL SARC-F, risk of sarcopenia	1.77	0.73-4.38 1	0.203	1.85	0.76-4.48 1	0.174
>4 <4 NRS-2002, nutritional risk	2.26	0.33-1.33 1	0.052	2.22	0.96-5.11 1	0.060
>3 <3 SGA, malnutrition	1.88	0.44-7.95 1	0.387	1.86	0.44-7.73 1	0.392
Present Absent Low BMI	4.47	1.06-18.7 1	0.040	4.18	1.01-17.2 1	0.047
Present Absent CC	1.28	0.57-2.90 1	0.542	1.22	0.54-2.76 1	0.631
Low Adequate Myopenia	1.17	0.53-2.56 1	0.688	1.27	0.57-2.83 1	0.553
Present Absent Myopenic obesity	1.06	0.47-2.38 1	0.878	1.14	0.50-2.60 1	0.746
Present Absent Myosteatosis	3.06	1.21-2.72 1	0.018	2.82	1.11-7.20 1	0.029
Present Absent	1.42	0.40-5.06	0.581	1.35	0.37-4.85	0.639

HR, hazard ratio.

* Cox regression adjusted for age, sex, and underlying disease.

In the univariate analysis of risks, all tools analyzed for the defi nition of malnutrition, nutritional risk, and risk of sarcopenia were associated with the incidence of death. Likewise, low BMI and CC reflected an increased risk of death, which was more common among patients with a low BMI and a low CC (< 33 cm for women and < 34 cm for men). Among the body composition components investigated, the combination of myosteatosis and myopenia (with or without visceral obesity) resulted in the greatest increase in the risk of death (Table 2).

The multivariate Cox regression analysis demonstrated that the type of hospital admission (clinical or surgical) was the only clinical predictor of mortality in our study sample, with a nearly three times greater likelihood of death in surgical patients (hazard ratio: 2.90; 95% CI: 1.28-6.54; *p* = 0.010). Among nutritional factors, malnutrition, as assessed with the SGA, and myopenic obesity were independent prognostic factors for death whether adjusted for confounding variables or not. Patients with each of those two conditions were, respectively, three and approximately four times more likely to die than were those without either condition (Table 3).

## DISCUSSION

A classification of malnutrition on the SGA was an independent predictor of mortality in our patient sample, which is in agreement with data described in previous studies^**(^22-24^)**^. Sánchez-Torralvo et al.^**(^25^)**^ analyzed a sample of hospitalized older adults with hip fractures and found a three to four times higher risk of mortality at three, six, and twelve months among patients classified as malnourished on the SGA in comparison with those classified as well-nourished. The items that compose the SGA reflect long-term changes, such as unintentional weight loss, a reduction in food intake, anorexia, and muscle loss, and are consequently predictors of morbidity and mortality^**(^22^)**^.

This study demonstrates the potential of myopenic obesity defined by CT to be a prognostic indicator of mortality in hospitalized patients. A growing body of evidence suggests that two main components of body composition (skeletal muscle and adipose tissue) have distinct characteristics and can impede the recovery of patients^**(^26^)**^.

The loss of muscle mass is widely recognized as being related to a variety of adverse outcomes in diseases, including cancer as well as cardiovascular, liver, and kidney disease^**(^26-28^)**^. In addition, obesity, which is characterized by excess adipose tissue, is a condition with metabolic effects that are harmful to health^**(^29,30^)**^. The world is currently experiencing a pandemic of obesity, and individuals with excess weight account for a significant portion of the hospitalized population. Therefore, the problem that emerges is how to identify muscle loss masked by adipose tissue in overweight or obese patients, given that, as demonstrated by our data, myopenia can be present in any BMI range.

Feng et al.^**(^26^)**^ consistently found that, among patients with cirrhosis followed for two years, survival was significantly lower in those with concomitant myopenia (defined by a low SMMI) and visceral obesity (determined by excess VAT). In the largest study conducted to date, Quint et al.^**(^28^)**^ found that myopenia, myosteatosis, and myopenic obesity were associated with mortality in a sample of 828 kidney transplant candidates.

Studies diverge with regard to the denomination of muscle mass deficit. In some, sarcopenia is mistakenly described as a low quantity of muscle mass. However, sarcopenia is defined as low muscle mass accompanied by a decline in functioning, which is not always assessed^**(^26-28^)**^. Therefore, we used the term myopenia, given that CT enables the assessment of muscle quantitatively and qualitatively but not functionally.

Another point of divergence that limits the comparison of our results with those of other studies involving myopenic (or sarcopenic) obesity resides in the diagnosis of obesity, whether the condition is determined on the basis of the BMI (> 30 kg/m^2^) or excess VAT. Our findings show that the capacity to predict mortality disappeared when obesity was defined by BMI. Feng et al.^**(^26^)**^ reported that using excess VAT for the determination of obesity enables identifying specific subgroups with worse outcomes to be identified, and that the prevalence of obesity can vary considerably as a function of the use of BMI or VAT.

In conclusion, the present study identified that individuals classified as malnourished based on the SGA and those with myopenic obesity were at greater risk of mortality. However, this study has limitations that should be considered. The small number of cases of myopenia may have affected the inference of the results. In addition, there is no global consensus regarding the cutoff points for myopenia and myosteatosis assessed by CT. The cutoff points used in this study were chosen because they are the most widely used, discussed and accepted in scientific investigations**(^6,27,28^)**.

This is the first study to explore changes in muscle and adipose tissue defined by CT and associations with mortality in a heterogeneous population of hospitalized patients. In contrast, previous studies examined patients with specific diseases^**(^6,26,28^)**^. The population in our study had a broad age range and was composed of patients with different comorbidities admitted to clinical or surgical wards with acute or chronic disease. Despite recent discoveries, there is a need for further studies to investigate the prognostic impact of body composition variables defined by CT.
